# Crosstalk between innate metabolism and gut microbiome

**DOI:** 10.1002/alz.088373

**Published:** 2025-01-03

**Authors:** Rima Kaddurah‐Daouk

**Affiliations:** ^1^ Department of Medicine, Duke University, Durham, NC USA

## Abstract

**Background:**

The GI tract is home to approximately 70% of the body’s immune cells, >100 million enteric neurons, and ∼40 trillion bacteria. This co‐localization of myriad immune, neural and bacterial cells creates complex interactions that regulate almost every tissue in the body, including the brain. Importantly, peripheral and GI inflammation occur in neurodegenerative diseases such as Parkinson’s disease (PD) and Alzheimer (AD) contributing to gut brain axis. The activation state and function of microglia, brain‐resident innate immune cells, has been implicated in AD mouse models and is regulated by the gut microbiome. Gut microbial changes are linked to development of neuropsychiatric diseases. These disorders, classically studied from a brain‐centric view and associated with neuroinflammation, often exhibit GI symptoms and peripheral inflammation.

**Method:**

In this session we will address metabolomics technologies applied by the Alzheimer Disease Metabolomics Consortium (ADMC) for the study of Alzheimer disease. State‐of‐the‐art metabolomics and lipidomics technologies combined with genomic and imaging data are used to map metabolic failures across the trajectory of the disease. Immune metabolomics platforms are being developed and used to link immune function changes and metabolic alterations in blood and in immune cells with link to cognition.

**Result:**

Our studies confirmed that peripheral metabolic changes influenced by the exposome and gut microbiome inform about cognitive changes, brain imaging changes, and ATN markers for disease confirming that peripheral and central changes are connected, in part through the metabolome. We defined metabolic differences between men and women with AD, developed initial brain metabolome for AD and defined lipidomics signatures that inform about mechanism of APOE ε2 resilience for AD. A major role for inflammation and immune dysregulation is noted and linked to signatures that inform about cognition and brain imaging changes. We will illustrate how we are applying immune metabolomics platforms to map cross talk between metabolism immune function inflammation and brain metabolic health.

**Conclusion:**

Metabolomics presents powerful tools to the study of inflammation, immune dysregulation, in AD pathogenesis.

